# Quantum Sensing for Real-Time Monitoring of Drug Efficacy in Synovial Fluid from Arthritis Patients

**DOI:** 10.1021/acs.nanolett.3c01506

**Published:** 2023-09-07

**Authors:** Arturo Elías-Llumbet, Yuchen Tian, Claudia Reyes-San-Martin, Alejandro Reina-Mahecha, Viraj Damle, Aryan Morita, Hugo C. van der Veen, Prashant K. Sharma, Maria Sandovici, Aldona Mzyk, Romana Schirhagl

**Affiliations:** † Department of Biomedical Engineering, University of Groningen, University Medical Center Groningen, Antonius Deusinglaan 1, 9713AW Groningen, The Netherlands; ‡ Laboratory of Genomic of Germ Cells, Biomedical Sciences Institute, Faculty of Medicine, University of Chile, 1027 Independencia, Santiago, Chile; § Department of Orthopaedic Surgery, University of Groningen, University Medical Center Groningen, Antonius Deusinglaan 1, 9713AW Groningen, The Netherlands; ∥ Department of Rheumatology and Clinical Immunology, University Medical Center Antonius Deusinglaan 1, 9713AW Groningen, The Netherlands; ⊥ Institute of Metallurgy and Materials Science, Polish Academy of Sciences, Reymonta 25, 30-059 Cracow, Poland

**Keywords:** diamonds, nanodiamonds, relaxometry, NV centers, arthritis, quantum sensing

## Abstract

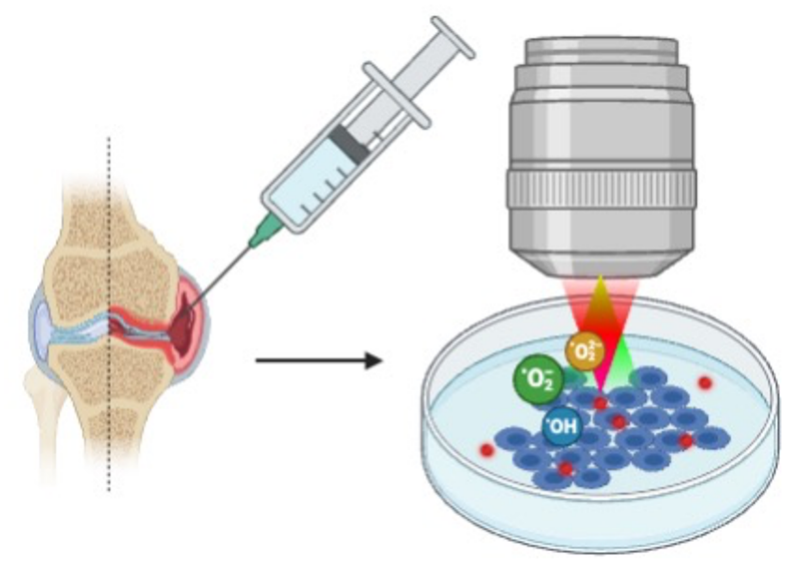

Diamond-based *T*_1_ relaxometry is a new technique that allows nanoscale magnetic resonance measurements. Here we present its first application in patient samples. More specifically, we demonstrate that relaxometry can determine the free radical load in samples from arthritis patients. We found that we can clearly differentiate between osteoarthritis and rheumatoid arthritis patients in both the synovial fluid itself and cells derived from it. Furthermore, we tested how synovial fluid and its cells respond to piroxicam, a common nonsteroidal anti-inflammatory drug (NSAID). It is known that this drug leads to a reduction in reactive oxygen species production in fibroblast-like synoviocytes (FLS). Here, we investigated the formation of free radicals specifically. While FLS from osteoarthritis patients showed a drastic decrease in the free radical load, cells from rheumatoid arthritis retained a similar radical load after treatment. This offers a possible explanation for why piroxicam is more beneficial for patients with osteoarthritis than those with rheumatoid arthritis.

Arthritis is a common disease that can lead to disabilities and a diminished quality of life. Multiple causes of arthritis require different treatment strategies. The two most common types of arthritis are osteoarthritis (OA) and rheumatoid arthritis (RA). OA is the most common joint disease worldwide, affecting an estimated 10% of men and 18% of women over 60 years of age.^1^ About 0.5–1% of the world population is currently affected by rheumatoid arthritis (RA).^2^ Different types of arthritis are characterized by differences in the type and level of inflammation (in RA more pronounced than in OA), the oxidative stress and compensating antioxidants produced by cells in the synovial fluid.^3,4^

Oxidative stress is caused by a variety of molecules, and especially free radicals (molecules with an unpaired electron) are challenging to detect.^5^ Several methods can be utilized for measuring radicals, the damage they cause,^4^ or certain responses to them at the DNA, RNA, or protein level.^6−8^ These methods are usually destructive and do not offer spatial resolution. There are also probes that convert into fluorescent molecules when they react with ROS.^9^ However, these reactions are irreversible. Thus, the probes reveal the sample’s history rather than the current state and bleaching limits their usability.^10^ Spin labels are another alternative for detecting radicals.^11,12^ They react with the radicals to form a stable derivative, which can be detected with conventional magnetic resonance imaging (MRI) or electron spin resonance (ESR). While these methods are widely used and even allow in vivo imaging, they are limited in sensitivity. As a consequence, they typically offer a spatial resolution in the millimeter range or down to a micrometer range at best.^13^

Diamond magnetometry is a method that may offer a solution to some of these issues. The technique is based on a fluorescent defect in a diamond, which changes its optical properties based on its magnetic surrounding.^14^ Since optical signals can be read out more sensitively than magnetic signals, the technique allows nanoscale magnetic resonance measurements. This technique is so sensitive that even the small magnetic signal of single electrons or a few protons can be detected.^15−17^

A specific type of diamond magnetometry called *T*_1_ relaxometry has the advantage that it requires only optical excitation and readout. *T*_1_ relaxometry has already been successfully used in physics.^18−20^ This method is sensitive to spin noise and is thus particularly suited to sensing radicals. This was demonstrated for studying aging in yeast cells^21^ and sensing mitochondrial activity in mural macrophages^22^ as well for studying free radical generation in cells which are impacted by viruses^23^ or bacteria.^24^ Here we explore the applicability of *T*_1_ relaxometry for measuring free radicals in clinical practice. We applied this method for the first time in samples from patients.

## Collecting Samples

Synovial fluid (SF) was collected from six patients with OA and six patients with RA undergoing primary total knee arthroplasty (TKA). Synovial fluid was extracted from the suprapatellar pouch prior to arthrotomy using a 10 mL syringe and a 16g needle. After harvesting, the sample was transferred to a sterile 120 mL sample container (Spectainer, SIMPC566, Avantor (VWR), The Netherlands) within the sterile area of the operation room. The sample was kept at room temperature until the end of the operation. Later, the sample was placed in an incubator under sterile conditions for a maximum of 30 min before the experiments.

## Free Radical Detection

To measure free radical generation, we performed a sequence of *T*_1_ relaxometry measurements. We excite NV centers with a laser pumping the NV centers into the bright ms = 0 state of the ground state. Over time the NV centers relax back into the darker equilibrium between the ms = 0 and ms = ±1 states. This process, which we can follow by collecting the fluorescence intensity, is faster in the presence of radicals.

These measurements were performed with a home-built diamond magnetometer which is commonly used in the field and described in a previous study^25^ and in the Supporting Information.

**Figure 1:**
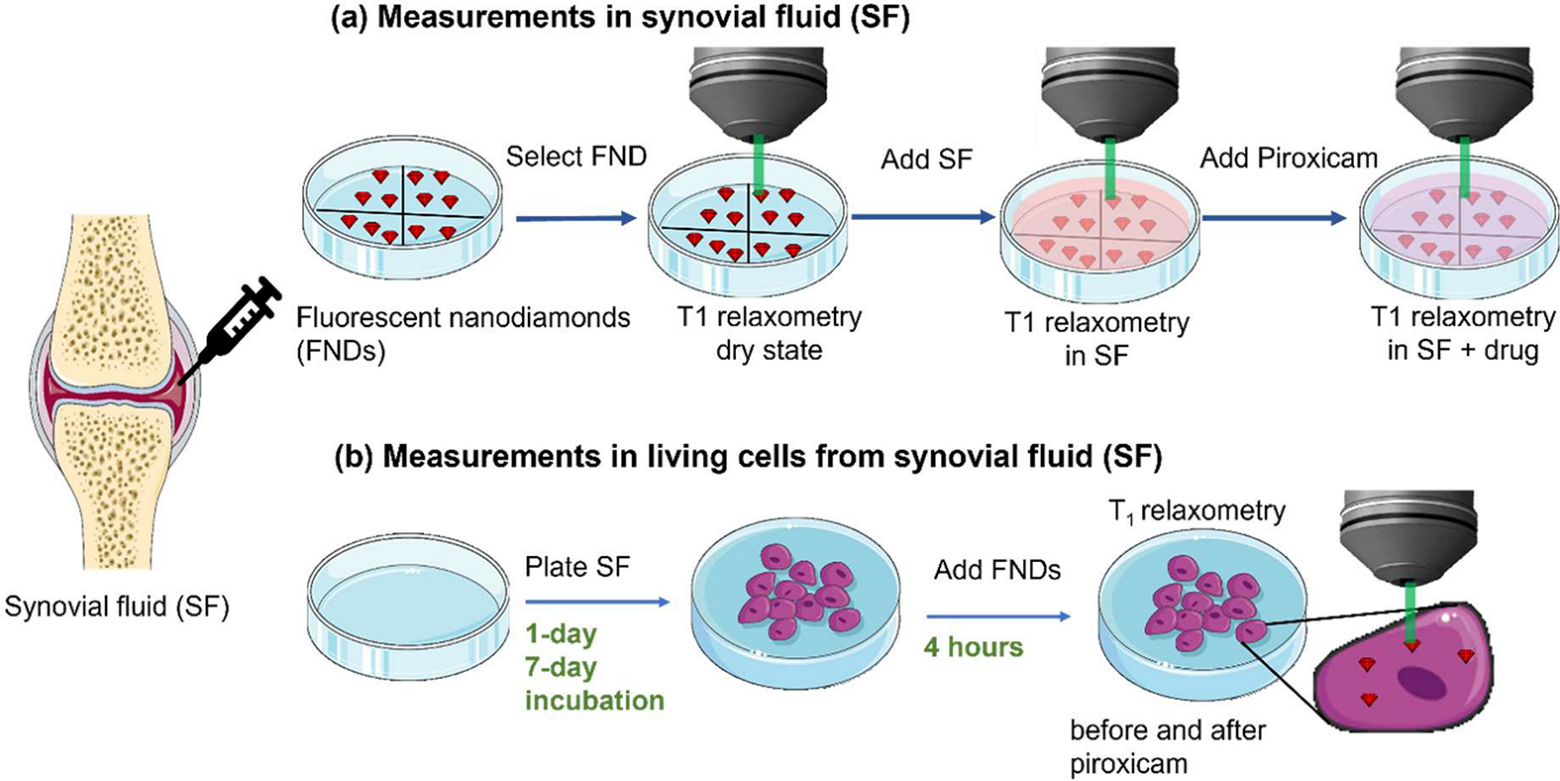
Schematic summary of the *T*_1_ experiments in this article: (a) measuring complete synovial fluid and (b) measuring in cells derived from synovial fluid.

The workflow of the performed experiments is shown in Figure 1 and is described in the following paragraphs.

## *T*_1_ Measurements in the Complete Synovial Fluid

Prior to the measurements, the suspension of FNDs was placed on 35 mm diameter Petri dishes (suitable for microscopy) and left to dry at RT, resulting in immobilization of nanoparticles at the dish surface. For every selected FND, we performed *T*_1_ measurements in a dry state for 30 min as an initial condition. Then we added 200 μL of complete synovial fluid (synovial fluid containing cells from synovium) and measured *T*_1_ for another 30 min for the same particle. Then, we added piroxicam to the synovial fluid at a final concentration of 2 μg/mL and performed *T*_1_ measurements for 30 min. Control measurements were performed to evaluate the interaction of FNDs with piroxicam and, therefore, its potential impact on the recorded *T*_1_ values. This *T*_1_ sequence was measured similarly as previously described, except that synovial fluid was replaced with PBS. Measurements in cells are described and shown in the Supporting Information.

## Data Analysis

All data are reported as mean value ± standard deviation (SD) with at least three independent repetitions. A statistical analysis of data was conducted using GraphPad Prism 8.0.1 software, and the significance was tested by a one-way ANOVA and Wilcoxon test.

**Figure 2:**
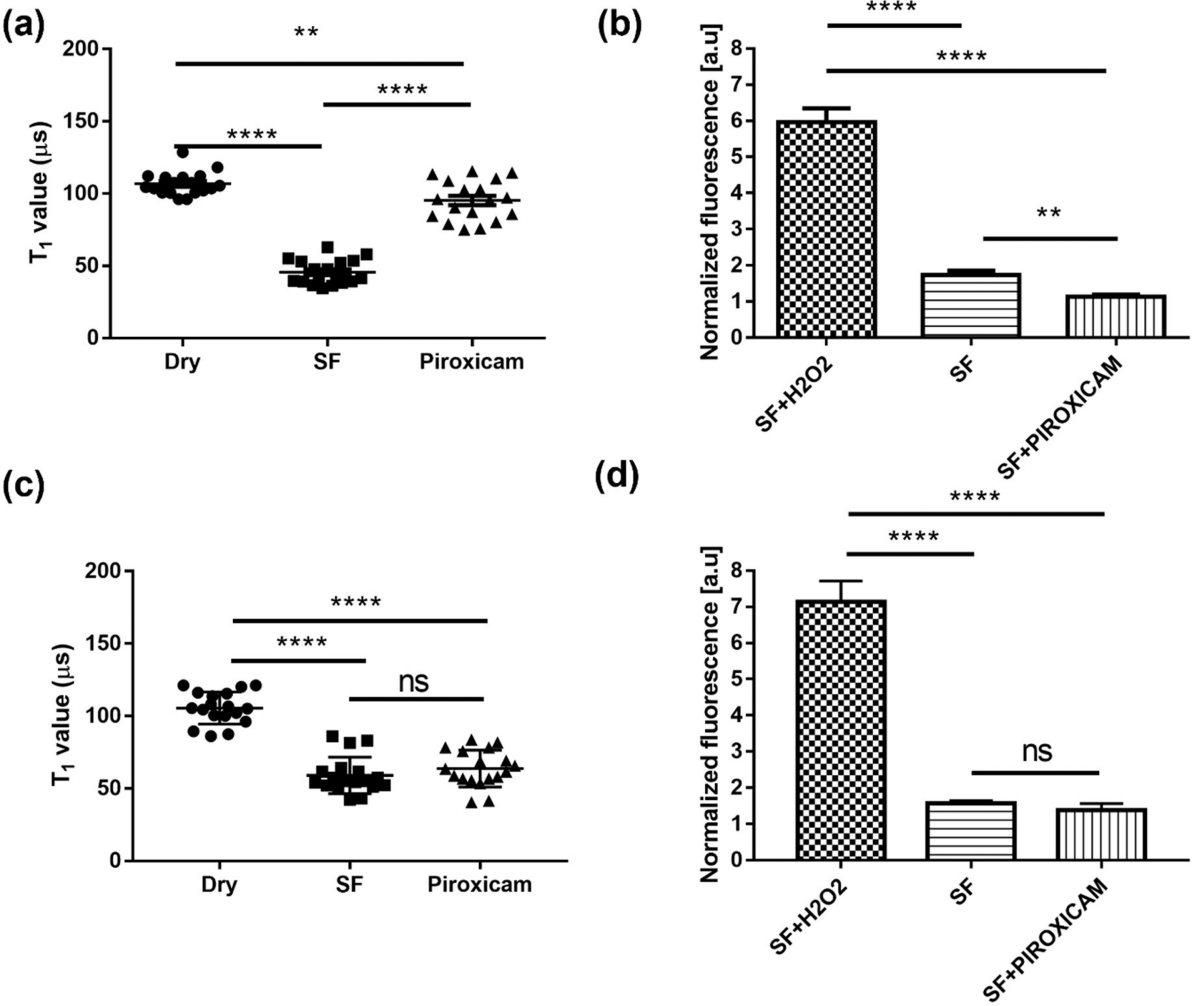
Radical versus ROS production in synovial fluid. (a) Relaxometry measurements revealing the local free radical load in the synovial fluid from osteoarthritis patients. (b) ROS production in samples from the same patients. (c, d) Results from free radical and ROS detection in synovial fluid from rheumatoid arthritis patients. The experiments were repeated three times for each patient (six OA and six RA patients), and error bars represent standard deviations (**p < 0.01, ****p < 0.0001).

Osteoarthritis and rheumatoid arthritis are characterized by different levels of inflammation with a higher presence of immune cells for the latter. However, we do not know if that means that we can expect their elevated radical level in the synovial fluid of arthritis patients or only in the cellular compartments. When we compare the changes in *T*_1_ after adding synovial fluid (see Figure 2) to a dish with dry nanodiamonds, we see a decrease in *T*_1_ value that indicates a significant level of free radicals in synovial fluid from both types of patients. However, we do not see a significant difference between them. Results obtained from the conventional DCFDA assay are also comparable. After measuring the initial radical levels, we added piroxicam and performed another *T*_1_ measurement on the same particle. While we observed a substantial decrease in radical formation in the osteoarthritis samples, the rheumatoid arthritis samples remained unperturbed. To exclude effects that piroxicam itself might have on the measurement, we performed a control measurement where we added piroxicam to FNDs on a glass surface in the absence of cells (see Figure S1 in the Supporting Information). In this case, we did not observe any changes in *T*_1_.

We also performed measurements on synovial-fluid-derived cells. As shown in Figure S3 in the Supporting Information, we observed trends in cells similar to those we have seen in the synovial fluid. The literature has reported that piroxicam (and NSAIDs in general) affects the production of reactive oxygen species by phagocytes.^26^ Piroxicam is more effective in treating osteoarthritis than rheumatoid arthritis.^27^ The fact that piroxicam reduces radical formation only in osteoarthritis samples might explain why. We observe the same trend in the conventional ROS assay. While the two methods provide similar results in this case, there are a few differences. Our technique can measure the current radical load while ROS probes reveal the history of the sample. It is possible to perform measurements on the same particle and location before and after an intervention. This way, a sample can function under its own control. This means that we can differentiate between the initial variability between cells and patients and the impact by the intervention. Since FNDs are only sensitive to radicals in their immediate surroundings, it is possible to obtain localized information from within a few nanometers from the particle surface.

## Cell in the Synovial Fluid

We performed flow cytometry experiments in order to identify which cell types we derived from OA and RA synovial fluid (see the Supporting Information).

**Figure 3:**
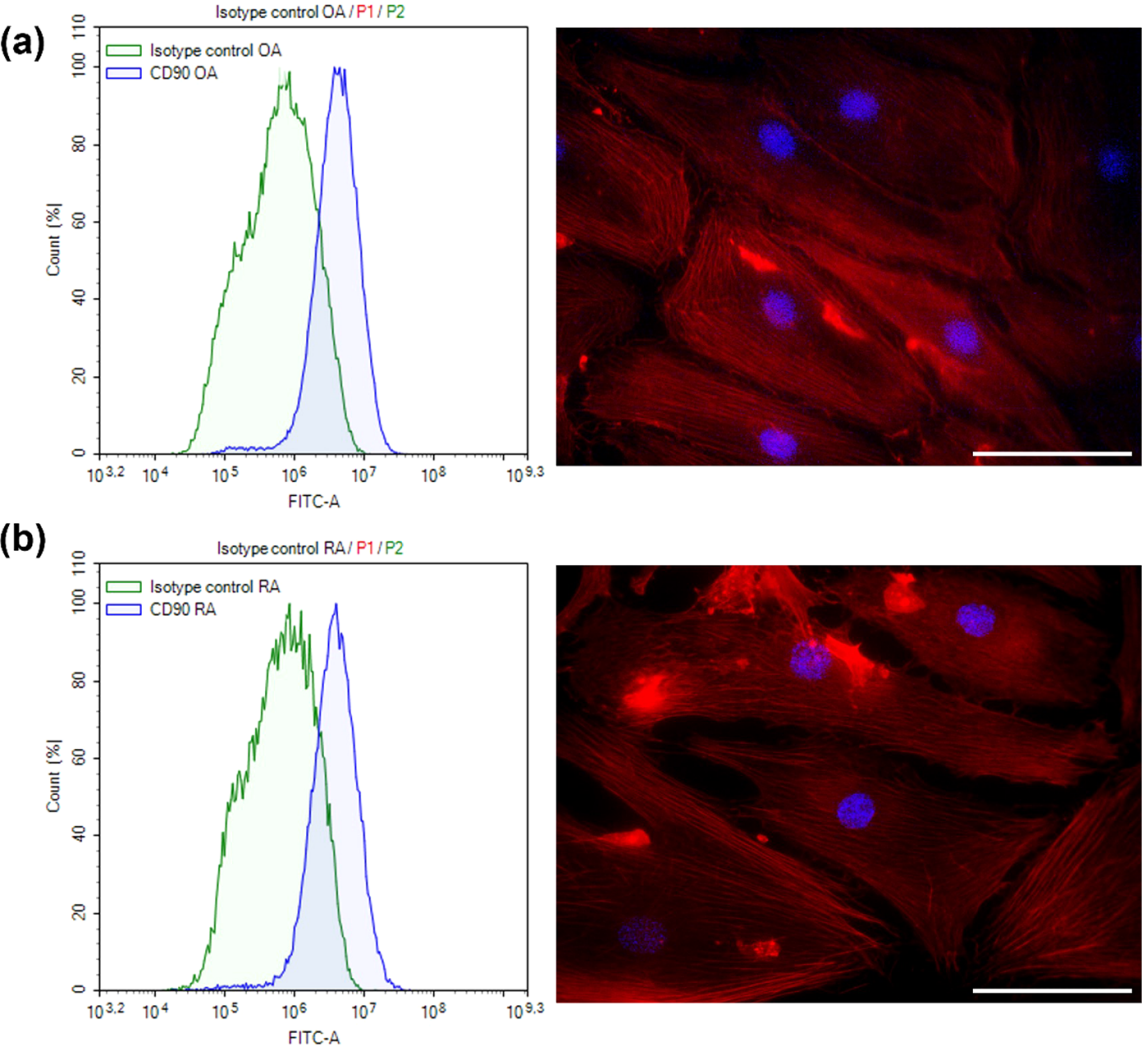
Fibroblast-like cells from synovial fluid of OA (a) and RA (b) patients based on the presence of the CD90 receptor and cell morphology. Histograms were normalized to the number of cells. The scale bar is 50 μm.

The morphology of both types of cultured spindle-shaped cells was characteristic for synoviocytes (see Figure 3a,b). As we can see in Figure 3, cells also express CD90 receptors, which indicate that they are synoviocytes.

De Sousa et al. have demonstrated that the mitochondrial activity of the OA and non-OA cells increases when exposed to OA, but not to non-OA synovial fluid.^28^ Stenfeldt et al. compared the effects of piroxicam on superoxide production by NADPH-oxidase, mediated by two closely related G-protein-coupled receptors expressed on neutrophils, the formyl peptide receptor (FPR) and the formyl peptide receptor-like 1 (FPRL1). They showed that piroxicam inhibits the neutrophil responses triggered through FPR but not through FPRL1. The inhibition mechanism was due to a reduced binding of the activating ligand to its cell surface receptor.^29^ As the formyl peptide receptor (FPR) is also present in the fibroblast-like synoviocytes, we speculate that cells derived from the OA and RA synovial fluid respond differently to piroxicam due to the expression of different variants of FPR receptors.

Here we demonstrate that diamond relaxometry can be used to correlate clinical efficacy with radical formation after treatment with Piroxicam. We only see a decrease in radical formation in osteoarthritis samples where the drug is more effective compared with samples obtained from the patients suffering from rheumatoid arthritis.

## Supporting Information Available

Control measurements for *T*_1_ in absence of cells, confirmation of the cell types by flow cytometry, experimental details of *T*_1_ measurements, and comparison with conventional ROS detection method (PDF)
