# Supplementary material for: Quantum Sensing for Real-Time Monitoring of Drug Efficacy in Synovial Fluid from Arthritis Patients
Source: Nano Lett. 2023 Sep 7;23(18):8406–10. doi: 10.1021/acs.nanolett.3c01506 (PMC10540259; doi:10.1021/acs.nanolett.3c01506)
Supplement: Supplementary file 1 — nl3c01506_si_001.pdf [file nl3c01506_si_001.pdf]

Supplementary information

Quantum sensing for real-time monitoring of drug efficacy in synovial fluid from arthritis patients

Arturo Elías-Llumbet1,2#, Yuchen Tian1, Claudia Reyes San Martin1, Alejandro Reina-Mahecha1, Viraj Damle1, Aryan Morita1, Hugo C. van der Veen3, Prashant K. Sharma1, Maria Sandovici4, Aldona Mzyk1,5#, Romana Schirhagl1**

1Department of Biomedical Engineering, University of Groningen, University Medical Center Groningen, Antonius Deusinglaan 1, 9713AW Groningen, the Netherlands

2Laboratory of Genomic of Germ Cells, Biomedical Sciences Institute, Faculty of Medicine, University of Chile, 1027 Independencia, Santiago, Chile

3 Department of Orthopaedic Surgery, University of Groningen, University Medical Center Groningen, Antonius Deusinglaan 1, 9713AW, Groningen, The Netherlands

4Department of Rheumatology and Clinical Immunology, University Medical Center Groningen, 9713AW, Groningen, The Netherlands

5Institute of Metallurgy and Materials Science, Polish Academy of Sciences, Reymonta 25, 30-059 Cracow, Poland

# these authors contributed equally
*corresponding authors
Email: romana.schirhagl@gmail.com
aldonamzyk@googlemail.com

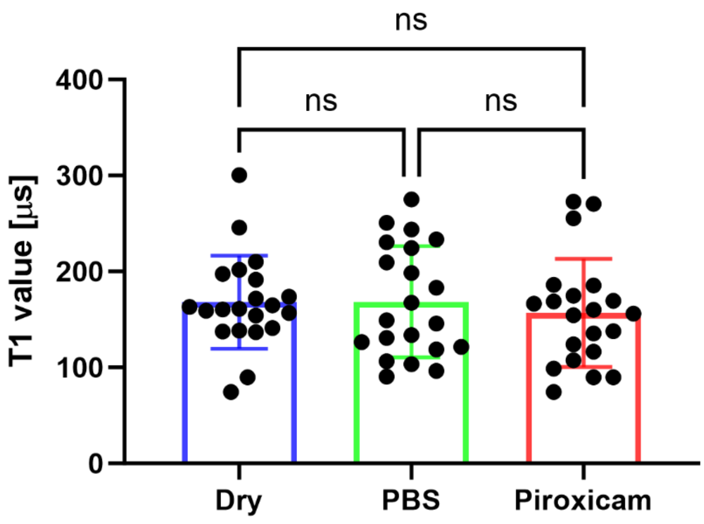

Supplementary Figure S1. Results of T1 relaxometry control measurements without cells or synovial fluid. The experiments were repeated 21 times for each step (dry, PBS, and Piroxicam) in the sequence, and error bars represent standard deviations.

Identification of cells from synovial fluid

Cells derived from synovial fluid were centrifuged for 10 min at 300 rcf. The cell pellet was resuspended in PBS containing 2% BSA to obtain a concentration of 1000 cells per ul. Immunostaining was performed using the following antibodies:

- Isotype control (IgG1 mouse anti-limpet, FITC, Cat. # 11-4714-42, eBioscience, 5 ul per 150 000 cells)
- CD33 (IgG1 mouse anti-human, FITC, Cat. # SAB4700155, Sigma-Aldrich, 2 ul per 150 000 cells)
- CD90 (IgG1 mouse anti-human, FITC, Cat. # SAB4700706, Sigma-Aldrich, 2 ul per 150 000 cells)
- CD3 (IgG1 mouse anti-human, FITC, Cat. # F0522, Sigma-Aldrich, 5 ul per 150 000 cells)
- CD11B (IgG1 mouse anti-human, FITC, Cat. # F2648, Sigma-Aldrich, 5 ul per 150 000 cells)
- CD86 (IgG1 mouse anti-human, FITC, Cat. # SAB4700146, Sigma-Aldrich, 10 ul per 150 000 cells)
- CD14 (IgG1 mouse anti-human, FITC, Cat. # SAB4700106, Sigma-Aldrich, 10 ul per 150 000 cells)

Antibodies were added to 150 ul of cell suspension in low-binding 1.7 mL vials (Cat. # 3207, Costar) and were incubated for one hour at RT, protected from light. Afterwards, 3 mL of pre-warmed 2% BSA in PBS were added to each vial and centrifuged for 10 min at 300 rcf. The pellet from each vial was resuspended in 500 uL of 4% formaldehyde and incubated at RT for 10 min. Then, the samples were centrifuged for 10 min at 300 rcf and resuspended in 150 ul of 2% BSA in PBS to proceed with flow cytometry within 30 min. The samples were transported at RT, and flow cytometry experiments were performed in a NovoCyte Quanteon cytometer (Agilent). The NovoExpress software version 1.5.0 was used for flow cytometry analysis.

A part of the cultured cells was fixed with 4% formaldehyde and stained with phalloidin-TRITC to visualize cell morphology (F-actin cytoskeleton). Images were taken with TissueFAXS microscope (63x/1.40 oil objective).

Samples from both OA and RA patients were negative for the expression of CD33, CD3, CD11b, CD14, and CD86, as indicated by the full overlap between the signal from these markers and the one from the isotype control (see supplementary Figure S2). Conversely, samples from RA and OA patients were highly positive for CD90 (see Figure 4 a, b). Even when there was some overlap between the CD90 signal and the one from the isotype control.

Fibroblast-like synoviocytes are important cellular components of the inner layer of the joint capsule. They can be found in both layers of the synovial membrane and contribute to normal joint function by producing extracellular matrix components. However, under inflammatory conditions, they may start to proliferate, undergo phenotypical changes, and become central elements of OA or RA.

Table 1 Interrogated cell markers and corresponding cell types.

Antigen	Cell types
CD33	myeloid progenitors, monocytes, granulocytes, dendritic cells, and mast cells
CD90	fibroblasts, activated endothelial cells, primitive hematopoietic stem cells (CD34+), fetal liver cells, and fetal thymocytes
CD3	T cells and some thymocytes
CD11b	granulocytes, monocytes, macrophages, dendritic cells, and NK T cells
CD86	activated B and T cells, monocytes, macrophages, dendritic cells, and astrocytes
CD14	monocytes, macrophages, granulocytes, dendritic cells

Source: Human Cell Differentiation Molecules (HCDM) → BioLegend

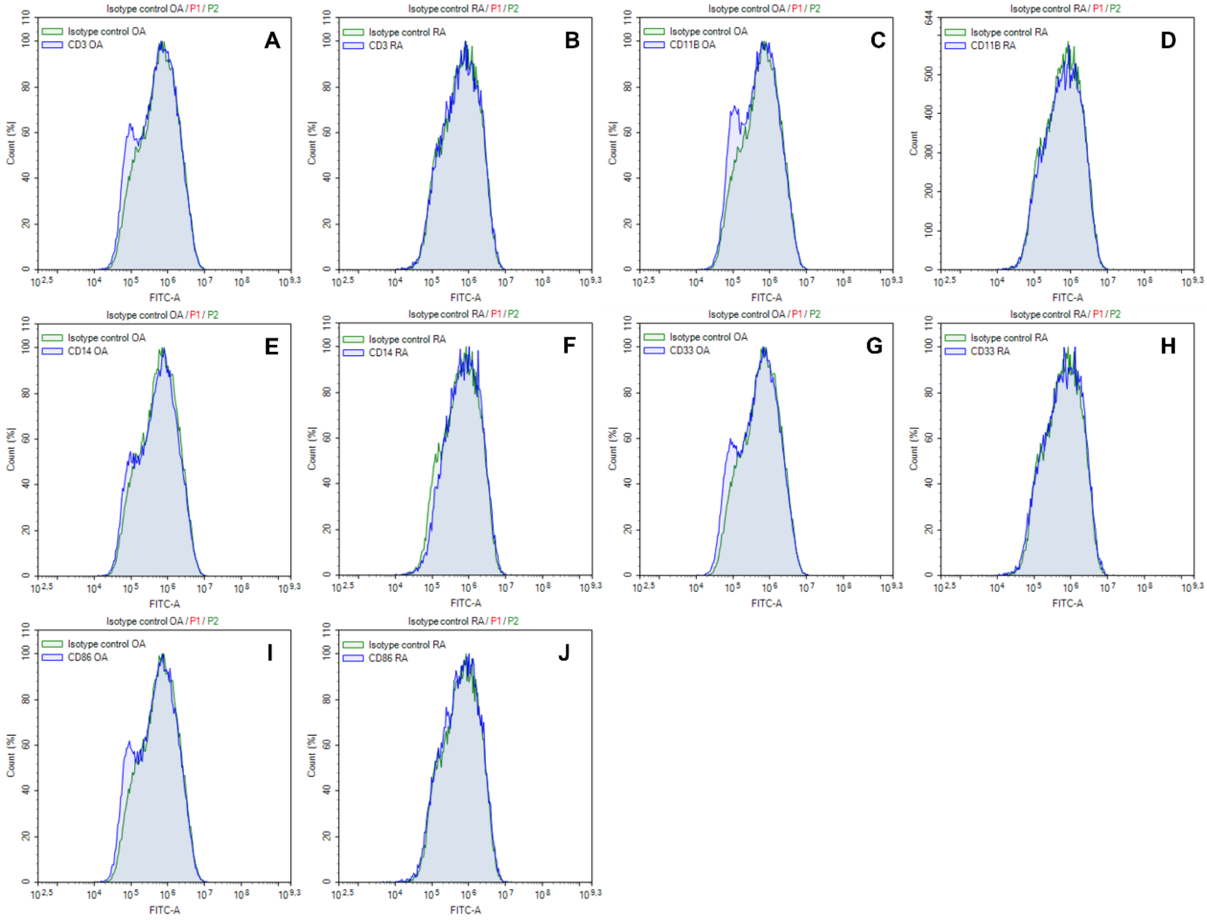

Supplementary Figure S2. Markers (CD3, CD118, CD14, CD33, CD86) with no detectable expression in cell cultures from synovial fluid of OA and RA patients. Samples from OA patients (A, C, E, G, I) and RA patients (B, D, F, H, J). Histograms were normalized to the number of cells.

Materials:

The fluorescent nanodiamonds (FNDs) in this work were commercially available particles from Adamas Nanotechnology (USA). These particles have a hydrodynamic diameter of 70 nm and are produced by grinding HPHT diamonds and irradiation with 3MeV electrons at a fluence of $5*10^{19}$ e/cm^2. After irradiation, they undergo high-temperature annealing. They contain an average of 500 NV centers (Nitrogen-Vacancy centers) per diamond (determined by EPR by the manufacturer) [[1]]. The final step of the production is a cleaning in oxidizing acids, which removes non-diamond carbon residues. As a result, the surface chemistry of these FNDs is oxygen-terminated. Previous literature has already characterized these particles extensively [[2],[3]]. Dulbecco's Modified Eagle's Medium (DMEM-HG) was provided by Sigma Aldrich (The Netherlands), Fetal Bovine Serum (FBS), penicillin and streptomycin trypsin/EDTA from Life Technologies (The Netherlands), and phosphate-buffered saline (PBS) from Invitrogen (The Netherlands). Piroxicam and phalloidin-TRITC was bought from Sigma Aldrich (The Netherlands). 2',7'-dichlorodihydrofluorescein diacetate (DCFDA) was purchased from Millipore Corp. (USA).

Ethical considerations:

The University Medical Center Groningen (UMCG) review board approved this study (14.07.2021 METc 2021/419 Magnetometry as a new tool to study joint inflammation), and all participants signed an informed consent.

Detection of reactive oxygen species

In order to evaluate the overall level of reactive oxygen species in collected samples, we have performed a DCFDA assay according to the manufacturer's recommendation and as shown earlier [4]. After entering the cell, DCFDA is deacetylated and later oxidized by ROS to 2,7-dichlorodihydrofluorescein (DCF), which is highly fluorescent. These fluorescence intensity changes were measured using a plate reader (excitation at 485 nm and emission at 535 nm) to determine cellular ROS production directly in the synovial fluid and in cultured cells with and without piroxicam treatment. The positive control was prepared by treating synovial fluid or cells with 25 µM H_2O_2.

Intracellular T1 measurements

The laser (532 nm, CNI, Changchun, China) was attenuated to 50 µW (2 × 105 W cm^{-2}) at the location of the sample (at continuous illumination). To collect the fluorescence, we used an Olympus UPLanSApo40× NA = 0.95 air objective. A 550 nm long-pass filter and a confocal pinhole are used to separate scattered laser light from fluorescence. For detection, we used an avalanche photodiode (SPCM-AQRF-15-FC) in single-photon counting mode. The NV centers were polarized with 5 µs long laser pulses intermitted by dark times (from 0.2 µs to 10 ms). To determine the polarisation state of the NV centers, we observed the fluorescence within the first 0.6 µs of each laser pulse. Since particles within cells are moving (and for drift correction in stationary particles), we also track the particle. To this end, the measurement is automatically paused every 5 seconds to perform a quick scan to find the particle and move the laser to the new location of the particle. While each individual measurement takes several microseconds, we usually repeat the measuring sequence about 10,000 times to obtain an excellent signal-to-noise ratio.

The collected synovial fluid (SF) was used to establish patient-specific cell cultures. For that purpose, the SF was centrifuged for 5 min at 1000 rpm. The pellet of cells was resuspended in a DMEM high glucose medium containing 10% FBS and 1% of penicillin-streptomycin. Cells were plated on glass-bottom Petri dishes. After one and seven days in culture, cells were incubated for 4 hours with 2 µg/ml of 70 nm FNDs resuspended in the medium. Afterwards, the samples were rinsed with fresh medium, and the initial T1 relaxometry measurements were performed. Next, the same cells were treated with Piroxicam at a final 2 µg/ml concentration. Then T1 measurements were performed using the same FNDs as in the initial measurements.

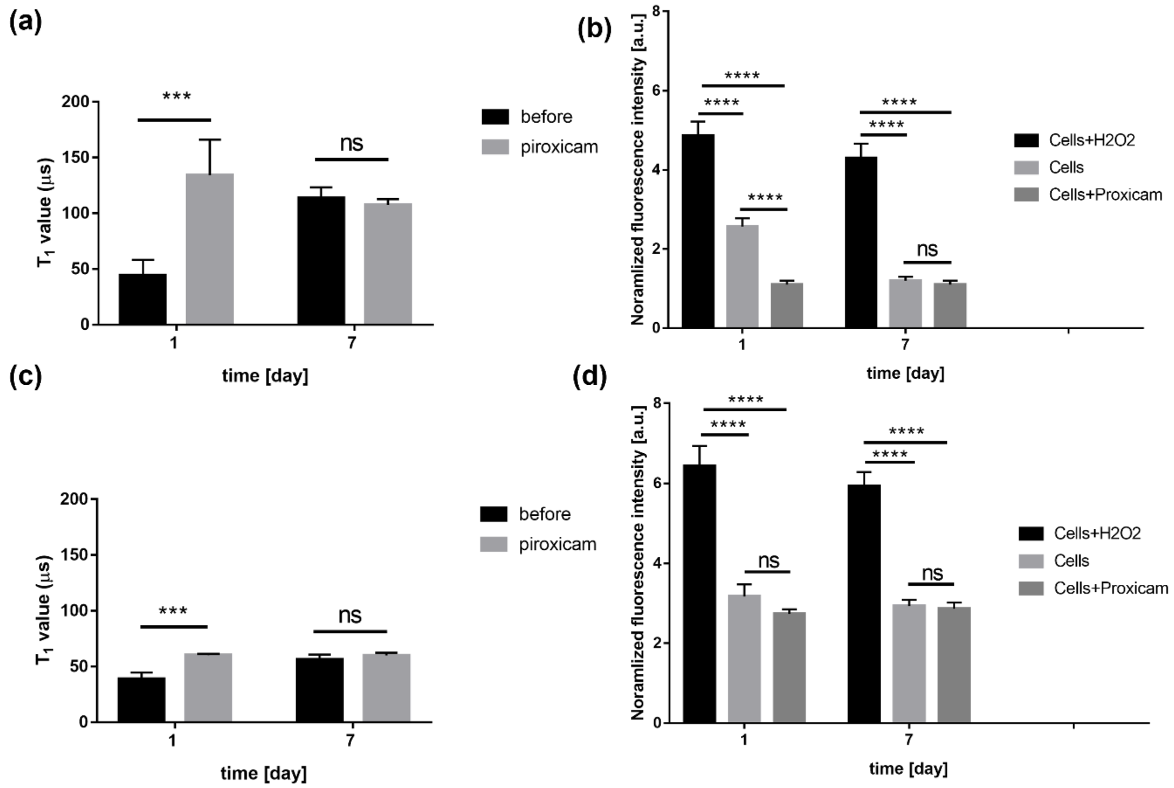

Supplementary Figure S3. Radical versus ROS production in cells derived from synovial fluid and cultured for 1 and 7 days. (a) The results of relaxometry measurements reveal the local free radical load in cells from synovial fluid of osteoarthritis patients. (b) depicts the ROS production in samples from the same patients. (c,d) show the results from free radical and ROS detection in cells from synovial fluid of rheumatoid arthritis patients. The experiments were repeated three times for each patient and error bars represent standard deviations. (*** $p < 0.001$, **** $p < 0.0001$).

---

[1] Shenderova, O.A., Shames, A.I., Nunn, N.A., Torelli, M.D., Vlasov, I. and Zaitsev, A., 2019. Synthesis, properties, and applications of fluorescent diamond particles. Journal of Vacuum Science & Technology B, Nanotechnology and Microelectronics: Materials, Processing, Measurement, and Phenomena, 37(3), p.030802.

[2] Ong, S.Y., Van Harmelen, R.J.J., Norouzi, N., Offens, F., Venema, I.M., Najafi, M.H. and Schirhagl, R., 2018. Interaction of nanodiamonds with bacteria. Nanoscale, 10(36), pp.17117-17124.

[3] Hemelaar, S.R., De Boer, P., Chipaux, M., Zuidema, W., Hamoh, T., Martinez, F.P., Nagl, A., Hoogenboom, J.P., Giepmans, B.N.G. and Schirhagl, R., 2017. Nanodiamonds as multi-purpose labels for microscopy. Scientific reports, 7(1), pp.1-9.

[4] Hemelaar, S.R., Saspaanithy, B., L'Hommelet, S.R., Perona Martinez, F.P., Van der Laan, K.J. and Schirhagl, R., 2018. The response of HeLa cells to fluorescent nanodiamond uptake. Sensors, 18(2), p.355.
